# The spin-dependent properties of silicon carbide/graphene nanoribbons junctions with vacancy defects

**DOI:** 10.1038/s41598-021-03363-z

**Published:** 2021-12-13

**Authors:** Golnaz Khanlar, Sahar Izadi Vishkayi, Hamid Rahimpour Soleimani

**Affiliations:** 1 Department of Physics, University Campus 2, University of Guilan, Rasht, Iran; 2 School of Physics, Institute for Research in Fundamental Sciences (IPM), P. O. Box 19395‑5531, Tehran, Iran; 3 Computational Nanophysics Laboratory (CNL), Department of Physics, University of Guilan, P. O. Box 41335‑1914, Rasht, Iran

**Keywords:** Nanoscience and technology, Physics

## Abstract

We have designed high-efficient spin-filtering junctions composed of graphene and silicon carbide nanoribbons. We have calculated the spin and charge transport in the junction by non-equilibrium Green’s function formalism combined with the density functional theory to find its spin-dependent electrical conductance, thermal conductance and Seebeck coefficient. In addition, the effect of Si and C atoms vacancies on the transport properties of the junction has been carefully investigated. The enhanced spin-filtering is clearly observed due to the edge and vacancy effects. On the other hand, vacancy defects increase the electrical and spin conductances of the junctions. The results show that the considered junctions are half-metal with reduced thermal conductance which makes them a suitable spin-dependent thermoelectric device. Our results predict the promising potential of the considered junctions for application in spintronic devices.

## Introduction

Finding and developing materials and devices with charge- and spin-dependent thermoelectric performance are attractive for many theoretical and experimental researchers in nanoelectronics and spintronics fields^1–5^. In fact, highly efficient charge-dependent thermoelectric materials can be used as the energy convertor from a waste thermal energy to a useful electrical form. In particular, it is possible to obtain a spin-dependent electrical potential by a thermal gradient in a device with a good spin-dependent thermoelectric efficiency^6^. Low dimensional materials, consists of different junctions, are promising candidates for this purpose because they showed high thermoelectric efficiency and also spin-filtering in previous studies^2–4,6,7^. Recently, a spin thermoelectric efficient device has been modelled by twisting the triangular π-dimer junctions^8^. Although single-molecular junctions are good candidates for high-efficient thermoelectric and spin-filtering devices^9^, one-dimensional junctions are interesting for their outstanding thermoelectric properties^2,3,5,6^. For example; it is theoretically predicted that a twisting bilayer graphene nanoribbon has a good thermoelectric efficiency^10^. Here, we introduce a junction with a high-efficient spin-filtering which is formed of silicon carbide (SiC) nanoribbon coupled to graphene nanoribbons. The spin-dependent transport properties of the junction are computed by density functional theory (DFT) in combination with Green function formalism. We have obtained the thermoelectric properties of the junction in the linear response regime^11^.

The combination of Silicon (Si) and Carbon (C) atoms in silicon carbide has caused the emergence of unique properties which attracted many researchers^12–14^. SiC nanoribbons have been synthesized via catalyst-free growth method which has honeycomb-graphene-like-structures^15^. There are many numbers of researches on SiC nanoribbons that show their interesting properties^13,15–17^. So, it can be a basic block for our investigation due to its unique properties. On the other hand, graphene is a highly flexible semi-metal with promising mechanical strength^18,19^ which can be coupled to other nanomaterials as the electrodes^20–22^. The graphene nanoribbon (GNR), has been proposed for the interconnect application. It indicated that both metallic and semiconducting GNRs provide lower resistivity than those of Cu and single-walled CNT (SWCNT) bundle^23^. The zigzag graphene nanoribbon is known as a popular material for spintronic investigation^24–27^. Because of the strong interface bonding between graphene and SiC^28^, we have selected zigzag GNRs as the electrodes which are coupled to the SiC ribbon.

An ideal thermoelectric device works very well if its heat conductivity is poor^29,30^. To obtain a suitable material for thermoelectric applications, it must have high electrical conductivity and the thermal conductivity should also be kept as low as possible to prevent heat loss. It is expected that in our considered junction, composed of SiC nanoribbon as the central region and GNRs as the electrodes, the phonon thermal conductance is small because of the reduction of phonon movement in inhomogeneous structures^31^, so it can be a suitable suggested thermoelectric device. On the other hand, the zigzag edges of GNR and SiC induce spin-dependent electrical properties in the device^16^ which make it a good spin-dependent thermoelectric device.

It is obvious that vacancy defects are created in the growth process of the structures. The vacancy defects affect the electronic and magnetic properties of SiC^17,32^. On the other hand, the vacancy defects reduce the thermal conductance of nanoribbons^33^ therefore we have considered the effect of SiC vacancy defect on the thermoelectric properties of the junction. Our results show that the Si- and C-defected SiC nanoribbons have a significant effect on the thermoelectric properties of the considered junction. Furthermore, there is a good spin-filtering in the junction which shows its potential for use as a spintronic device.

In the following, the computational methods are presented in “Computational methods” and the simulation results and discussions are given in “Result and discussions”. Finally, in “Conclusion”, a brief of the results is reported.

## Computational methods

**Figure 1:**
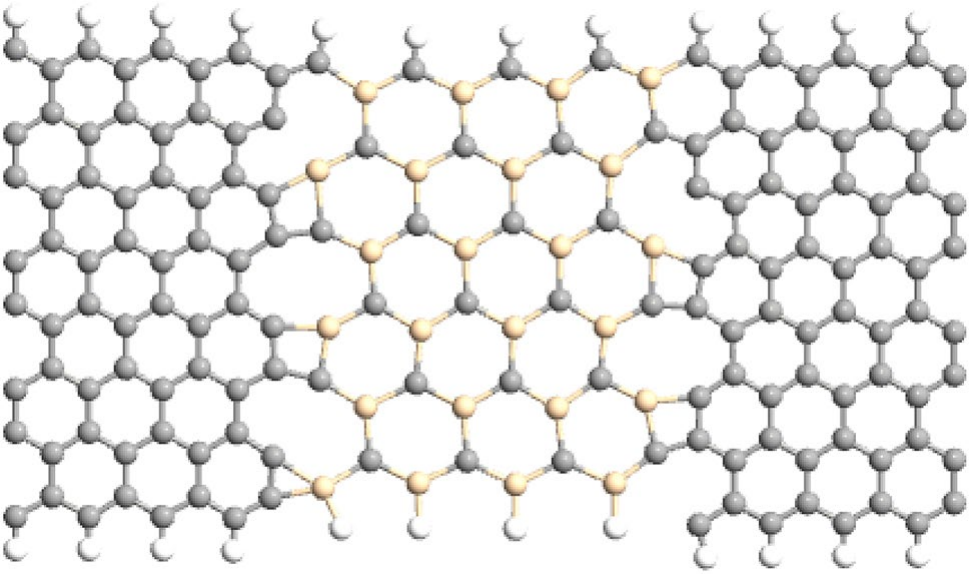
The optimized geometry of the silicon carbide nanoribbon between the GNRs. Carbon (silicon) atoms are represented by gray (yellow) circles*.*

The considered junction is shown in Fig. 1, which is composed of a silicon carbide nanoribbon (SiCNR) coupled to graphene nanoribbons (GNRs) in two sides as the electrodes. The GNRs and SiCNR with zigzag edges are considered to have extremely high magnetization at the edges. The length of central area is 9.778 (Å) and its width is 14.260 (Å). The size of central region is small enough to have ballistic transport in the junction. All the calculations for optimization of the structure had been done by SIESTA DFT-based package^34^. We had relaxed the atomic position of the considered device until the atomic force on each atom became less than 0.02 eV/Å. The stability of graphene nanoribbons^35,36^ and stable nanoribbons of SiC sheets^37–40^ had reported in the previous literatures. So, we have expected that our considered systems are stable enough to form. In order to understand the stability of the systems, we used a simple model and calculated the formation energy of the devices by ${E}_{F}={E}_{tot}-({N}_{C}{E}_{C}-{N}_{H}{E}_{H}-{N}_{Si}{E}_{Si})$, where ${N}_{C}, {N}_{H}$ and ${N}_{si}$ are the numbers of carbon, hydrogen and silicon atoms in the devices. The energy of free C, H and Si atoms are denoted by ${E}_{C}, {E}_{H},$ and ${E}_{Si}$ and the total energy of the device is given by ${E}_{tot}$ which is obtained by DFT calculations. We have obtained formation energy equal to − 6217.3 eV for the device with any vacancy, it is equal to − 1103.6 eV for C-defected SiC and − 1101.5 eV for Si-defected SiC. The mines amount of formation energy shows that these kinds of devices can be stable and the most stable device is a perfect channel without vacancy.

The transport properties of considered structures are calculated by Green function formalism which is implanted in TRANSIESTA package^41^. The converged cutoff energy for the high-level energy of wave function is 80 Hartree.

In SIESTA package, the effective potential is determined and the Hamiltonian matrix elements are calculated by standard algorithms^34^ in which the Kohn–Sham Hamiltonian and electron density of the device are calculated self-consistently. The Kohn–Sham Hamiltonian is given by

1 $$H=-\frac{{\hslash }^{2}}{2m}{\nabla }^{2}+{V}_{eff}\left[n\right]\left(r\right)$$

where the kinetic energy of the electron is described by the first term and the second term is the density, n, functional of the effective potential. Here, the effective potential consists of norm-conserving Troullier–Martins pseudopotentials^42^, generalized gradient approximation (GGA)^43^ as the exchange–correlation functional and Hartree potential which is electron density functional and obtained by Poisson equation^41^.

The charge (spin) thermoelectric efficiency of a system is obtained by the charge (spin) figure of merit, $Z{T}_{C\left(S\right)}$^6,11^

2 $$Z{T}_{C(S)}=\frac{{G}_{e(S)}{S}_{C(S)}^{2}T}{\left({\kappa }_{e}+{\kappa }_{ph}\right)}$$

where T is the absolute temperature of the junction. The electrical conductance and the electron thermal conductance are equal to ${G}_{e}=1/2({G}_{\uparrow }+{G}_{\downarrow })$ and ${\kappa }_{e}=1/2({\kappa }_{e\uparrow }+{\kappa }_{e\downarrow })$, respectively. The spin conductance is given by ${G}_{s}=1/2({G}_{\uparrow }-{G}_{\downarrow })$ where the spin-dependent electrical conductance, ${G}_{\sigma }$, and electron thermal conductance, ${\kappa }_{e\sigma }$ are respectively given by

3 $${{G}_{\sigma }= e}^{2}{L}_{0\sigma }$$

4 $${\kappa }_{e\sigma }= \frac{1}{ T}\left[{L}_{2\sigma }-\frac{{L}_{1\sigma }^{2}}{{L}_{0\sigma }}\right]$$

The charge (spin) Seebeck coefficient, ${S}_{C(S)}$, is the sum (mines) of spin-up and spin-down Seebeck coefficients given by

5 $${S}_{C(S)}=- \frac{1}{2\left|e\right|T}\left(\frac{{L}_{1}\uparrow }{{L}_{0}\uparrow }\pm \frac{{L}_{1}\downarrow }{{L}_{0}\downarrow }\right)$$

The integral coefficients of ${L}_{n\sigma }$ is obtained by

6 $${L}_{n\sigma }=\frac{1}{\hslash }\int d\varepsilon {T}_{\sigma }\left(\varepsilon \right)\left(-\frac{\partial f}{\partial \varepsilon }\right){\left(\varepsilon -\mu \right)}^{n}$$

where $f(\varepsilon )$ is the Fermi–Dirac distribution function of the left or right electrodes and $\mu$ is the chemical potential of the electrode. The spin and energy dependent transmission coefficient is given by^41,44^

7 $${T}_{\sigma }\left(\varepsilon \right)=Tr\left[{\gamma }_{L\sigma }\left(\varepsilon \right){\mathrm{G}}_{\sigma }^{R}\left(\varepsilon \right){\gamma }_{R\sigma }\left(\varepsilon \right){\mathrm{G}}_{\sigma }^{A}\left(\varepsilon \right)\right]$$

where ${\mathrm{G}}_{\sigma }^{R}\left(\varepsilon \right) ({\mathrm{G}}_{\sigma }^{A}\left(\varepsilon \right))$ expresses the retarded (advanced) Green function^6^ with spin $\upsigma$ and the broadening function of $\alpha$ electrode is

8 $${\gamma }_{\alpha \sigma }(\upvarepsilon )=\mathrm{i}[{\Sigma }_{\mathrm{\alpha }}\left(\varepsilon \right)-{\Sigma }_{\mathrm{\alpha }}^{\dagger}\left(\varepsilon \right)]/2$$

It describes the broadening of the energy levels due to coupling of the left (right) electrode to the central region and the self-energy matrix can be given by^41^

9 $${\Sigma }_{\alpha }\left(E\right)=\left[V {g}_{\alpha }\left(E\right) {V}^{+}\right]$$

where V is a matrix for describing the coupling between the sites of the device and the surface of electrode. ${g}_{\alpha }\left(E\right)$ denotes to the unperturbed retarded Green function of $\alpha$ electrode. The elements of coupling matrix, V, and unperturbed Green function formalism are obtained self-consistently by density functional theory based calculations. More details of the methods used in TRANSIETA package can be found in Refs.^41,45,46^.

The phonon thermal conductance, ${\kappa }_{ph}$, is given by^47^

10 $${\kappa }_{ph}=\frac{{\mathrm{\hbar }}^{2}}{2\pi {k}_{B}{T}^{2}}\underset{0}{\overset{\infty }{\int }}d\omega {\omega }^{2}{T}_{ph}\left(\omega \right)\frac{{e}^{\frac{\mathrm{\hbar }\omega }{{k}_{B}T}}}{{\left({e}^{\frac{\mathrm{\hbar }\omega }{{k}_{B}T}}-1\right)}^{2}}$$

where $\mathrm{\hbar }\omega$ is the phonon energy and ${T}_{ph}\left(\omega \right)$ is the phonon transmission coefficient^47^ calculated by Green function formalism in a way similar to the calculation of electron transmission coefficient. However, to find ${T}_{ph}$, we used reactive force field classical potential^48^ which makes the force constant matrices of the electrodes and the central region.

## Result and discussions

**Figure 2:**
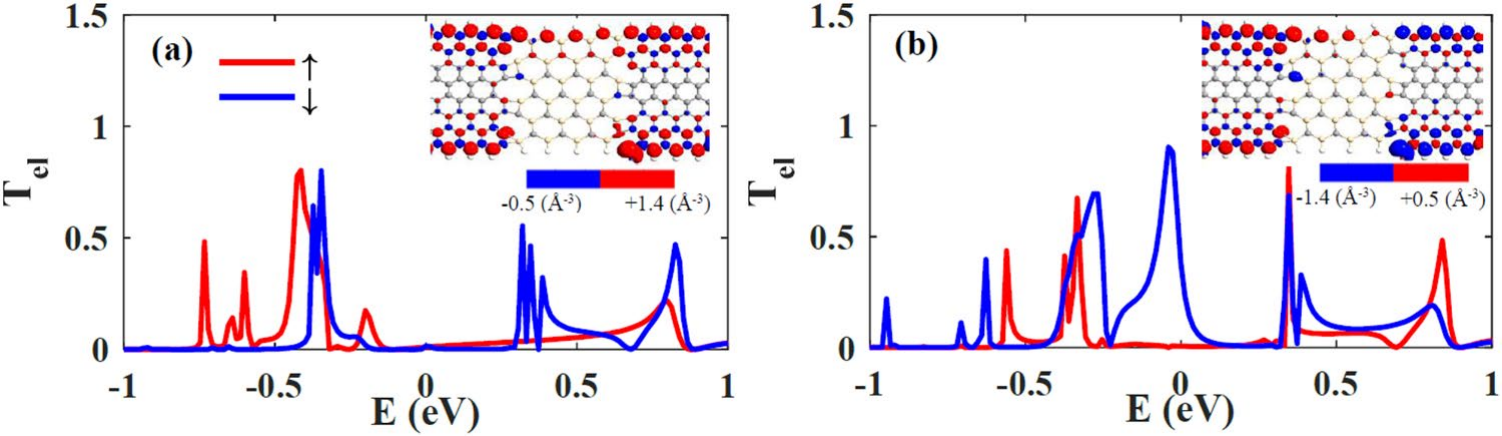
The spin-up (red lines) and spin-down (blue lines) electron transmission coefficients of SiCNR in (**a**) P and (**b**) AP configurations. The insets show the spin density diagram of the junction. Red (blue) color represents the spin-up (-down) density and the mean values of spin densities are given in the scroll bar at the bottom of the diagrams*.*

In this article, the edges of GNRs electrodes are considered in two spin configurations; parallel (P) and anti-parallel (AP), in which the spin orientations of carbon atoms at the edges of left and right electrodes are parallel and anti-parallel, respectively. In the SiCNR coupled to the electrodes with P configuration, the spin-up and spin-down transmission coefficients (${T}_{el\uparrow } and {T}_{el\downarrow }$) of carriers are obviously separated (see Fig. 2a). In the energy range of 0.05–0.25 eV, there is a small transmission for carriers with spin-up orientation, while spin-down carriers are not transmitted through the channel so we can consider that the device acts like a half-metal. It is interesting that half-metallicity is very intense in the AP spin-configuration of the electrodes because ${T}_{el\downarrow }$ is more than ${T}_{el\uparrow }$ around the Fermi energy and the spin-splitting is completely observed in Fig. 2b.

**Figure 3:**
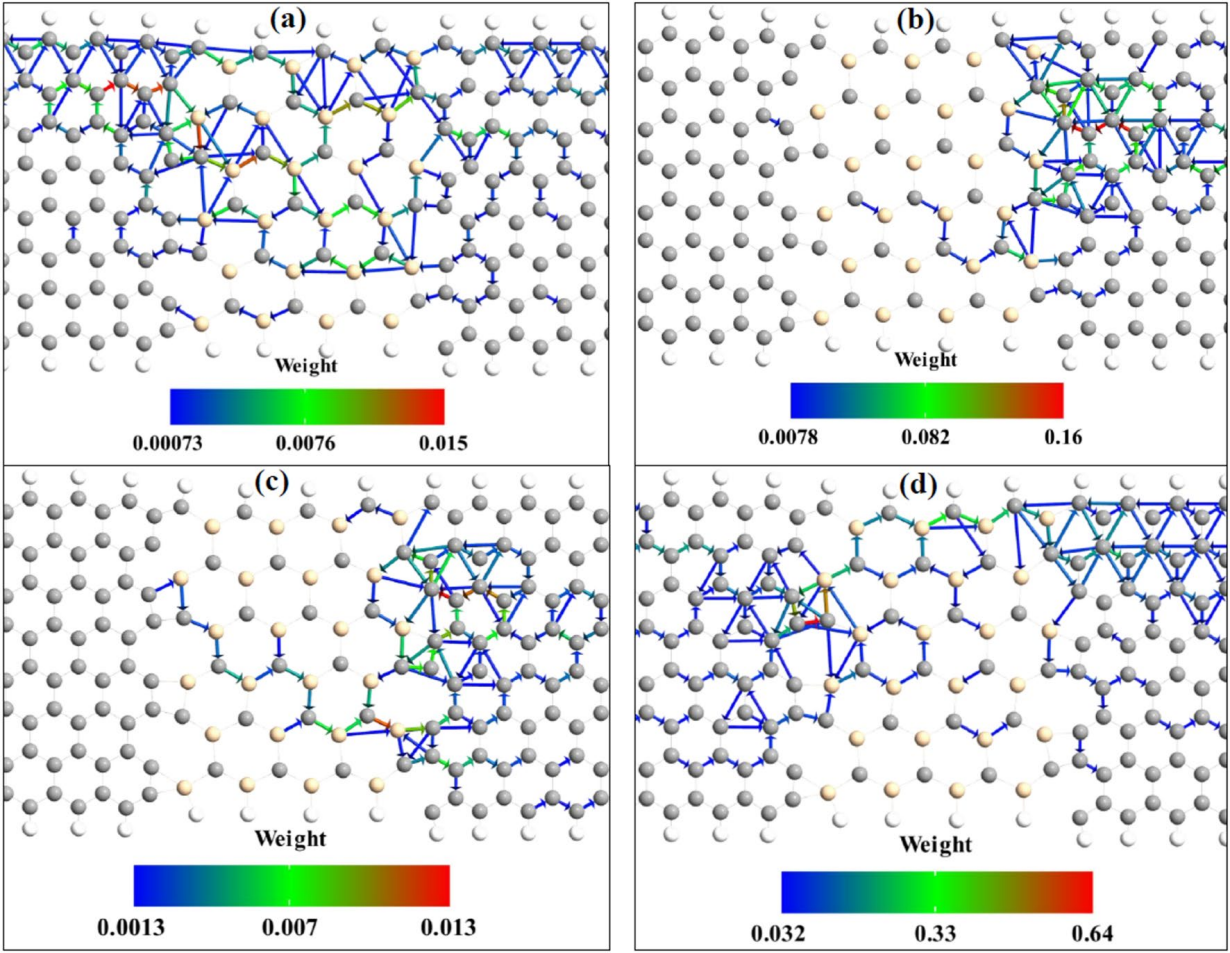
The spin-up (**a** and **c**) and spin-down (**b** and **d**) electron transmission pathway of SiCNR junction in P (**a** and **b**) and AP (**c** and **d**) configurations. Forward and backward arrows show the direction of transmission pathway to the right or left and the weight of the transmission pathway between the atoms are represented by the color according to the color bar.

In General, the electron transmission of spin-up and spin-down carriers are the same in common AP systems, but here a strong spin separation is observed due to the geometrical antisymmetric which exists in the central region. There are carbon (silicon) atoms at the top (bottom) edge of SiCNR. In the insets of Fig. 2a and b, it is obviously observed that the spin density of carbon atoms is more than silicon one in the central region of the junctions. Therefore, spatial separation of spin density is achieved. In P configuration, the spin-down energy states of the central region cannot transfer through the spin-up carrier states of the electrodes but in AP configuration one of the electrodes has spin-down energy states, so the spin-down electrons can transfer through the spin-down states of the junction and a maximum of transmission at zero energy is observed for the spin-down carriers. It is visible that the spin density of silicon carbide nanoribbon of the channel is dependent on the polarization of electrodes especially at the interface of SiCNR and ZGNR (see insets of Fig. 2). In P configuration, the spin density diagram shows that the carbon atoms have spin-up configurations at the edge of SiCNR like ZGNRs electrodes. On the other hand, at the interface of SiCNR and left ZGNR, there is an atom with a small spin-down density which leads to the creation of a small spin-down transmission through the device and the spin-up electrons are transmitted through the carbon atoms of SiCNR edge. Both spin-up and spin-down transmission pathway weights is ignorable in P configuration which is shown in Fig. 3a and b. It shows that there are not enough energy levels at the channel to let electrons pass through it. So the transmission spectrum is near to zero in the vicinity of zero chemical potential in the P configuration of electrodes. In the AP configuration of electrodes, the polarization of carbon atoms at the edge of SiCNR is decreased (see inset of Fig. 2b). This means that the spin-up electron transmission is less than the one in the P configuration. At the interfaces of SiCNR and ZGNR the spin-down density is increased which leads to the increment of spin-down transmission spectrum in the device which is also shown by the transmission pathway diagram in Fig. 3c and d. The bigger weight of the spin-down transmission pathway in comparison to the ignorable transmission pathway of spin-up electrons show the emergence of spin-filtering in the AP configuration. In other words, in AP configuration, the rearrangement of spin-density in the SiCNR leads to the creation of spin-down energy levels in the channel which allow the spin-down electrons to transfer through them while spin-up electrons are filtered.

**Figure 4:**
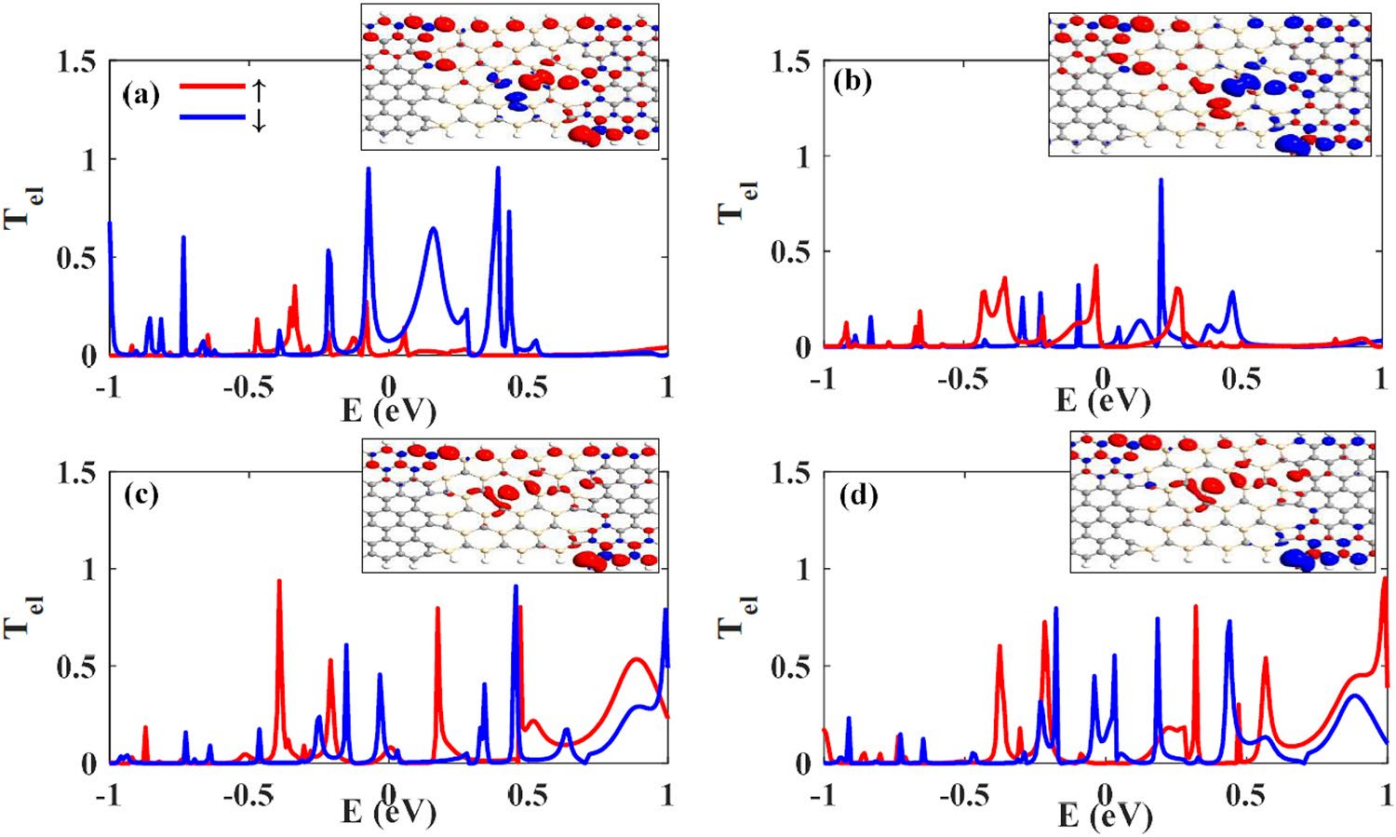
The spin-dependent electron transmission coefficients of (**a**) and (**b**) Si-defected SiCNRs in P and AP configurations and (**c**) and (**d**) C-defected SiCNRs in P and AP configurations, respectively. The insets show the corresponding spin density diagram of the junctions. Red (blue) color represents the spin-up (-down) density and the mean values of spin densities are as the same as Fig. 2.

Vacancy defects have an important role on the electrical and magnetic properties of structures. Here, we have considered the SiCNRs with vacancies at the channel. The vacancy leads to the increment of the spin density by electron injection because of the appearance of dangling bonds. In the Si-defected SiC, which a Si atom is eliminated at the center of the channel, the transmission coefficient is increased around the Fermi level; however, the transmission coefficient of spin-down carries is more than spin-up ones when the electrodes are in P configuration (see Fig. 4a), while in the AP configuration, Fig. 4b, ${{T}_{el}}_{\uparrow }$ is more than ${{T}_{el}}_{\downarrow }$ near the zero energy. In the P configuration of electrodes, the spin density diagram shows that the elimination of a Si atom changes the spin density of the pristine device (in comparison with the insets of Figs. 2a and 4a). The spin density has been moved to the central region (on the neighbors of the vacant atom) while it was focused on the upper edge of the pristine SiCNR. It is observed that the spin density is different on the nearest-neighbor carbon atoms at the left and right sides of the vacant atom. This kind of spin induction is due to extra electrons injection by the vacant atom. In the Ap configuration, the spin density diagram is changed at the edge of SiCNR and half of the carbon atoms at the edge of SiCNR have spin-down density. The carbon on the right hand of vacant atom is coupled ferromagnetically to the right electrode and two other nearest-neighbor carbon atoms (at the left and bottom of the vacant atom) are coupled anti-ferromagnetically to the first carbon atom in both P and AP configurations. In P configuration, the spin densities of the nearest-neighbour atoms of vacancy are vice versa of the spin densities of the same atoms at AP configuration. In other words, the sign of their spins comes from the interaction of the channel with the right electrode. As a consequence, in P configuration, two carbon atoms with spin-down electrons, around the vacancy, induce more spin-down transmission channels in the junction in comparison with the perfect SiCNR which can be observed in the transmission coefficient graph around the zero energy. On the other hand, in AP configuration, the mentioned carbon atoms have spin-up carriers, so ${T}_{el\uparrow }$ should be increased in the zero energy.

To find a comprehensive insight into the vacancy’s effects on the properties of the junction, we have also considered the elimination of C atoms at the middle of SiCNR and named it by C-defected SiC. The transmission coefficient of C-defected SiC is shown in Fig. 4c and d for P and AP configurations, respectively. By the elimination of a C atom at the center of the channel, its Si neighbors get dangling bonds that lead to injection of spin-density on them, but the spin-density of silicon atom is less than carbon atoms, therefore the edge carbon atoms are also the main source of spin density at the central region of the considered structures in both P and AP configurations. So, we expect to see a small deviation from the pristine configurations for transmission coefficients. The spin-up transmission is increased around the zero energy for P configuration. On the other hand, in AP configuration, the spin-down transmission is decreased around zero energy. Half-metallicity is also conserved in AP configuration of C-defected SiC.

**Figure 5:**
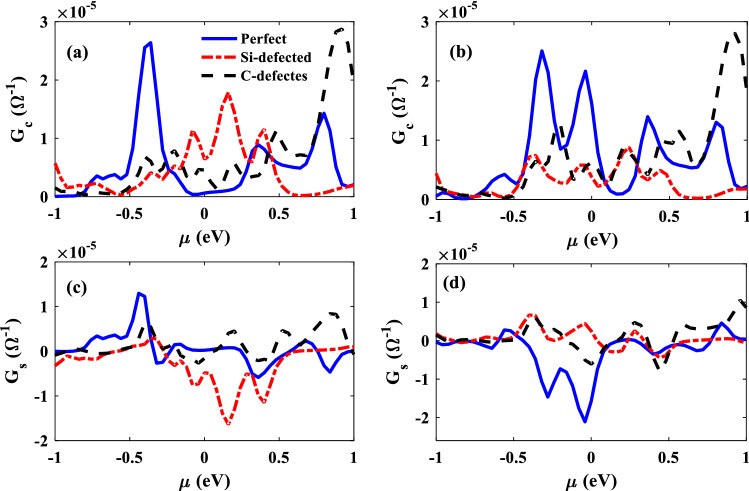
The electrical conductance, ${\mathrm{G}}_{\mathrm{e}}$, and the spin conductance, ${\mathrm{G}}_{\mathrm{s}}$, of the perfect (solid lines), Si-defected (dot-dashed lines), and C-defected (dashed lines) of junctions as a function of chemical potential for P (**a** and **c**) and AP (**b** and **d**) electrodes configurations*.*

The spin-filtering is observed in the transmission coefficients and spin-density diagrams of the considered junctions. Therefore, the spin-dependent thermoelectric properties of the junctions may be interesting for researchers. In this part, we explore the thermoelectric properties of the considered junctions. In Fig. 5a and b, the electrical conductances of the considered junctions are shown for P and AP configurations, respectively. It is obviously observed that the electrical conductance is increased for defected junction around the zero energy for P electrodes, because of the increment of the electron transmission coefficients which had been discussed previously. The electrical conductance is obtained by integrating over the transmission coefficient, so both of them have similar behavior. In Ap configurations, at the zero energy, we have observed that the amount of transmission is decreased in the presence of Si or C vacancies and as a consequence, the electrical conductances of the defected junctions are less than the perfect one in zero chemical potential. It should be noticed that this order is changed in another chemical potential because different energy levels play their own role on the electron transmission by the variation of chemical potential.

The spin conductance of the P configuration is shown in Fig. 5c. In μ = 0, the Si-defected SiC has the maximum value of G_s_ which is in agreement with the difference between ↑ and ↓ transmission coefficient of the junction. In Fig. 5d, for AP configurations, the maximum amount of G_s_ belongs to the perfect junction because of the large difference between spin-up and spin-down transmission coefficients of the perfect junction. Interestingly, the sign of G_s_ is vacant-type dependent. The positive value is obtained for Si-defected junction while C-defected junction has negative G_s_ value. In fact, ${T}_{el\uparrow }$ is more than ${T}_{el\downarrow }$ in Si-defected junction and it is vice versa in C-defected junction. So, the desired spin-conductivity can be achieved in the considered junctions by vacancy engineering.

**Figure 6:**
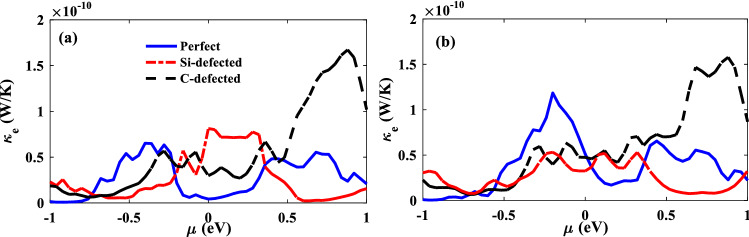
The electron thermal conductance of perfect (solid lines), Si-defected (dot-dashed lines), C-defected (dashed lines) of junctions as a function of chemical potential for P (**a**) and AP (**b**) electrodes configurations*.*

In Fig. 6a and b, the electron thermal conductance of the carriers is plotted in P and AP configurations, respectively. The electrons with different spin orientations carry thermal energy during the transport. Then the electron thermal conductance graph is similar to the electrical conductance one. Around zero energy, Si-defected junction has the highest values of electron thermal conductance and C-defected junction is in the second position while the electron thermal conductance of perfect junction is less than the others actually like the electrical conductance of P configurations (see Fig. 5a). In AP configurations, the electron thermal conductance is decreased by vacancy defects, because of the reduction of transmission coefficient of the AP junction in the presence of vacancy defects.

**Figure 7:**
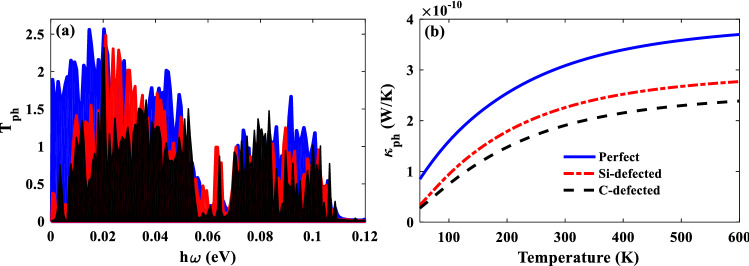
(**a**) The phonon transmission coefficient and (**b**) the phonon thermal conductance of the perfect (blue solid lines), the Si-defected (red dot-dashed lines) and the C-defected (black dashed lines) of junctions as a function of temperature*.*

In two-dimensional materials, like graphene, phonons are the most important quasi-particles to carry thermal energy. Therefore, we have calculated the phonon transmission coefficient and phonon thermal conductance of the junctions. It is clear that the phonon transport in P and AP junctions are the same. In Fig. 7a, it is observed that the phonon transmission coefficient values are decreased by vacancy and as a consequence, the phonon thermal conductance of the defected junction is less than the perfect junction (see Fig. 7b). To obtain a high-efficient thermoelectric device, we need to decrease the thermal conductance of the junction which is possible by applying vacancy defect. The phonon thermal conductance of the junctions is dependent on the temperature. It is observed that the thermal conductance is increased by increasing the temperature due to the increment of the thermal energy which is carried by phonons in higher temperatures. It should be noticed that despite the importance of electron–phonon interactions in large systems^49^, we have ignored the scattering process in our calculations. The size of SiCNR/ZGNR junctions is small enough to consider ballistic phonon and electron transport individually through the channel.

The Seebeck coefficient is the most important term in the figure of merit formula. The zero values of Seebeck coefficient show that the chemical potential of electrodes is equal to an energy level or it is placed exactly between two energy levels and as a consequence, both electrons and holes are carried in the junction at the same weight. On the other hand, the sign of ${S}_{c}$ has important information about the kind of carriers; if ${S}_{c}>0$, the electrons are the main carriers passed through the junction while holes are responsible for the transport when the values of charge Seebeck coefficients are negative.

**Figure 8:**
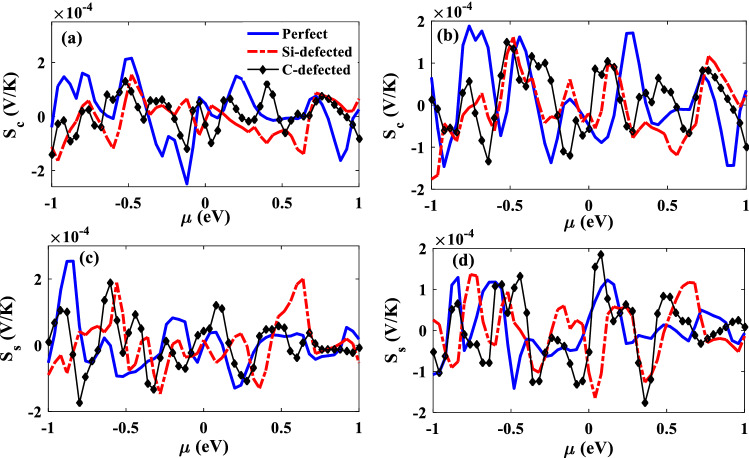
The charge Seebeck coefficient, ${\mathrm{S}}_{\mathrm{c}}$, and the spin Seeebeck coefficient, ${\mathrm{S}}_{\mathrm{s}}$, of the perfect (solid lines), Si-defected (dot-dashed lines), C-defected (dashed lines) junctions as a function of chemical potential for P (**a** and **c**) and AP (**b** and **d**) electrodes configurations*.*

In Fig. 8a and b, the charge Seebeck coefficient of the junctions are given in P and AP configurations. In P configuration, the positive ${S}_{c}$ value is achieved for the perfect junction at zero chemical potential, which shows the electrons are the main carriers. By vacancy defect, the carriers are changed to holes and the charge spin Seebeck coefficient is negative for both Si-defected and C-defected junction in P configuration. It is clearly in agreement with the transmission coefficient peaks which placed in negative energy near to zero energy (see Fig. 4). At zero chemical potential, ${S}_{c}$ is less than zero for all the junctions in AP configurations (Fig. 8b), so holes are the main carriers passed through the junctions. By variation of the chemical potential, different energy levels enter in the transport process then electrons or holes can be the charge carriers in the junction which make positive or negative charge Seebeck coefficient.

The difference between ${S}_{c\uparrow }$ and ${S}_{c\downarrow }$ gives the spin Seebeck coefficient that shows the junction ability for inducing a spin accumulation with a temperature gradient^6^. The values of the spin-dependent Seebeck coefficient are dependent on the strength of making spin accumulation in the junction while the negative (positive) sign shows that the spin-down (spin-up) Seebeck coefficient is more than the spin-up (spin-down) one. In Fig. 8c and d, the spin Seebeck coefficient of the P and AP junctions are shown. It is demonstrated that ${S}_{s}$ has an oscillating behavior as a function of chemical potential which is due to the entering of different spin states of different energy levels in the transport mechanisms. In zero chemical potential, there is a small difference between ${S}_{c\uparrow }$ and ${S}_{c\downarrow }$ of perfect P junction, which makes a small spin Seebeck coefficient. By applying vacancy defects, this difference amount is increased especially for C-defected junction. In AP configuration, despite the perfect junction has a positive spin Seebeck coefficient, the junctions with vacancies have negative values at $\mu =0$. This means that the spin accumulation type is changed by vacancy defect.

**Figure 9:**
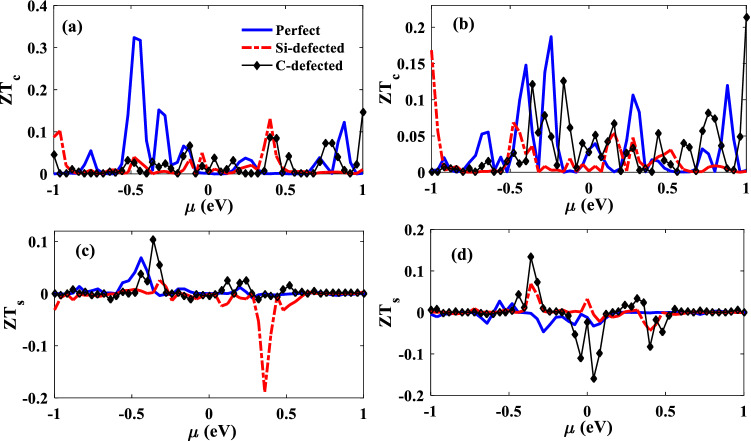
The charge thermoelectric efficiency, ${\mathrm{ZT}}_{\mathrm{c}}$, and the spin thermoelectric efficiency, ${\mathrm{ZT}}_{\mathrm{s}}$, of the perfect (solid lines), Si-defected (dot-dashed lines) and C-defected (lines with diamonds) junctions as a function of chemical potential for P (**a** and **c**) and AP (**b** and **d**) electrodes configurations.

By increasing the electrical conductance and Seebeck coefficient and decreasing the thermal conductance, the thermoelectric efficiency of the device is increased. In Fig. 9a, $Z{T}_{c}$ values of the perfect junction are ignorable around zero chemical potential because of vanishing the electrical conductance while applying vacancy defects increases its values. In $\mu =+0.04 eV$ and $\mu =-0.04 eV$, the $Z{T}_{c}$ values of C-defected and Si-defected junctions are around 0.05. By variation of the chemical potential, thermoelectric efficiencies of the defected junctions are increased up to $Z{T}_{c}=0.1$ which is a result of the increment of the Seebeck coefficient. In the junctions with AP configurations, around zero chemical potential, the perfect junction has non-zero $Z{T}_{c}$ values because of having the strong transmission channel in zero energy, and by changing the chemical potential, it also reaches $Z{T}_{c}=0.2$ (see Fig. 9b). In general, the C-defected junction is a better thermoelectric efficient device in comparison with the Si-defected junction because of its reduced phonon thermal conductance. The enhanced charge and spin thermoelectric efficiency of graphene nanoribbons^2,3,50^ and twisting bilayer graphene nanoribbons^10^ had been also reported. In our considered junctions, the amount of ZT_c_ is less than the monolayer and bilayer pristine graphene nanoribbons^10,50^ due to larger phonon thermal conductance, so we have shown that the defects can reduce the phonon thermal conductance which leads to the increment of $Z{T}_{c}$. On the other hand, in the SiCNR/ZGNR junctions, the spin-filtering leads to the creation of $Z{T}_{s}$ that are explained in the following.

In Fig. 9c and d, it is observed that the values of $Z{T}_{s}$ are increased by vacancy defect for both P and AP configurations, so the considered defects can induce a spin-dependent electrical potential difference in the junctions. Therefore, these junctions can be a promising candidate for the application in spintronics and logic memory devices. In fact, it is possible to make a desired electrical potential difference with a determined spin orientation, by engineering the chemical potential and vacancy defect type in the junction. For example, In the P configuration, at $\mu =-0.36 eV$, the amount of $Z{T}_{s}$ is equal to + 0.10 which shows the accumulation of spin-down carriers in the junction. On the other hand, $Z{T}_{s}$ is equal to − 0.18 at $\mu =+0.36 eV$ which shows that the junction is an efficient device for the creation of spin-down electrical potential difference. In AP configuration, the C-defected SiC has the best spin-thermoelectric efficiency, because its $Z{T}_{s}$ is about + 0.14 at $\mu =-0.35 eV$ while it is equal to − 0.16 at $\mu =+0.04 eV$. Therefore, a switchable spin-dependent electrical potential is obtained by changing the chemical potential in the C-defected junction.

## Conclusion

Here, a junction of SiC nanoribbon coupled to the zigzag GNRs is investigated in both parallel and antiparallel spin configurations of zigzag GNRs edges. The thermoelectric properties of the junction are calculated by density functional theory and Green’s function technique. Spin-polarized transmission coefficients are obtained for both P and AP configurations while a perfect half-metallic behavior is observed in the AP configuration due to the asymmetric edges of SiC in the central region. The Si- and C-defected SiC nanoribbons are also considered as the central region. The spin density of the junction is dependent on the edge and the type of vacancy defects in the junction. The results show that it is possible to engineer the sign of spin conductance by the type of vacancy defects. On the other hand, the vacancy defects leading to the reduction of phonon thermal conductance of the junction. In Seebeck coefficient analysis, it is demonstrated that the kind of charge carriers can be tuned by the type of vacancy defects. The spin Seebeck coefficient amount and sign are changed by the vacancy in the SiC region and the edge configurations of the GNRs. Our results suggest that the considered junctions can act as suitable spin filters in spintronics and logic memory devices. We have also predicted a switchable spin-dependent electrical potential in the C-defected junction with an acceptable spin thermoelectric efficiency.
